# Acoustic hologram-enabled simultaneous multi-target blood-brain barrier opening (AH-SiMBO)

**DOI:** 10.1038/s44172-025-00428-z

**Published:** 2025-06-02

**Authors:** Xinya Yao, Xiangkun Piao, Shulong Hong, Chenyu Ji, Mingyu Wang, Yan Wei, Zhouyang Xu, Jia-Ji Pan, Yanbo Pei, Bingbing Cheng

**Affiliations:** 1 Translational Research in Ultrasound Theranostics Laboratory, School of Biomedical Engineering, ShanghaiTech University, Shanghai, China; 2 State Key Laboratory of Advanced Medical Materials and Devices, ShanghaiTech University, Shanghai, China; 3 Institute of Modern Optics, School of Physics, Harbin Institute of Technology, Harbin, China; 4 Key Laboratory of Micro-Nano Optoelectronic Information System of Ministry of Industry and Information Technology, Harbin Institute of Technology, Harbin, China; 5 Key Laboratory of Micro-Optics and Photonic Technology of Heilongjiang Province, Harbin Institute of Technology, Harbin, China

**Keywords:** Biomedical engineering, Acoustics, Drug delivery

## Abstract

Focused ultrasound-induced blood-brain barrier (BBB) opening enables targeted brain drug delivery. However, achieving simultaneous multi-target BBB opening across various depths and regions remains challenging and cost-prohibitive. Here we address these challenges by employing encoded acoustic holograms combined with a single-element plane-wave transducer to generate precise focused acoustic fields. The holograms designed using an iterative angular spectrum approach and fabricated via 3D printing produce single or multiple foci at different depths and regions, which are confirmed by simulations and hydrophone-based measurements. Beam steering capability is demonstrated and further validated in vivo. We design a hologram with less than 10% variation in amplitude across different foci and successfully achieve biplane multi-target ( ≥3) BBB opening in the bilateral hippocampus and medial septum of mice in a single sonication session with no adverse effects. This innovative acoustic holographic approach for simultaneous multi-target BBB opening is time-efficient and cost-effective, presenting broad potential applications in brain drug delivery and neuromodulation.

## Abstract (web summary)

Xinya Yao and colleagues report an acoustic holographic approach for simultaneous arbitrary-plane multi-target opening of the blood-brain barrier. This time-efficient and cost-effective approach offers a promising strategy for sophisticated brain drug delivery and neuromodulation.

## Introduction

Focused ultrasound (FUS) is an emerging non-invasive treatment modality that has been widely used in both clinical and preclinical studies^1,2^. This technique can induce either temporary or lasting changes in tissue through thermal^3^ or non-thermal mechanisms^4^, enabling a range of therapeutic outcomes^5^. FUS holds tremendous clinical potential for drug delivery^6^, neuromodulation^7,8^, thermal ablation^9^ and immunomodulation. The combination of FUS and microbubbles allows for non-invasive, localized, transient, and safe opening of the blood-brain barrier (BBB) for brain drug delivery^6,10^. This technology has been proven effective and safe in rodents, non-human primates, and humans^11–15^. Recent studies have demonstrated that targeted BBB opening in the hippocampus, entorhinal cortex, frontal lobe and parietal lobe reduces β-amyloid burden while preserving cognitive function^6,16^. However, due to the rapid clearance of microbubbles from circulation (~5 min post-bolus injection), conventional multi-target FUS BBB opening typically requires sequential sonication of individual brain regions, necessitating multiple microbubble injections. Therefore, simultaneous multi-target BBB opening is highly desired for treating conditions involving large or anatomically distributed brain volumes, such as neurodegenerative diseases and gliomas^17,18^, as it enables BBB opening in a single sonication session. This approach reduces microbubble dosage, shorter anesthesia duration, and minimizes risks associated with repeated FUS exposures. Consequently, the demand for convenient and cost-effective methods to achieve simultaneous multi-target BBB opening is growing to enhance therapeutic efficacy and reduce treatment duration. Although phased-array systems offer the capability for simultaneous multi-target BBB opening, they present several limitations, such as high system complexity, energy loss, the need for phase correction, and high cost^19,20^. These challenges underscore the necessity for time-efficient and cost-effective alternative methods.

In recent years, substantial progress has been made in the design of acoustic metasurfaces for ultrasound beam focusing^21–23^. The acoustic hologram is a structure designed to reconstruct complex acoustic fields by spatially modulating the phase distribution of a transmitted wavefront. It can achieve precise control of reflection^24–26^ or transmission^27,28^ of wavefront at sub-wavelength scale^29^. Acoustic holograms have been demonstrated to generate ultrasound fields of any shape through the skull^22^, facilitating applications in bilateral BBB opening in both mice and non-human primates^30–32^. However, these studies only achieved symmetric two-target BBB opening on a single plane. Additionally, there has been a lack of systematic investigation of the focusing properties of acoustic holograms. The iterative angular spectrum approach (IASA) has emerged as a highly effective method for designing acoustic holograms, particularly when handling complex phase information^33^. Compared to the time-reversal approach^34^, IASA refines both phase and amplitude distributions through iterative optimization^35^. Furthermore, IASA is less constrained by the computational limitations or training data availability compared with the deep learning-based approach^36^. Therefore, IASA is a promising approach for the acoustic hologram design with the goal of achieving simultaneous multi-target BBB opening at arbitrary planes.

**Fig. 1:**
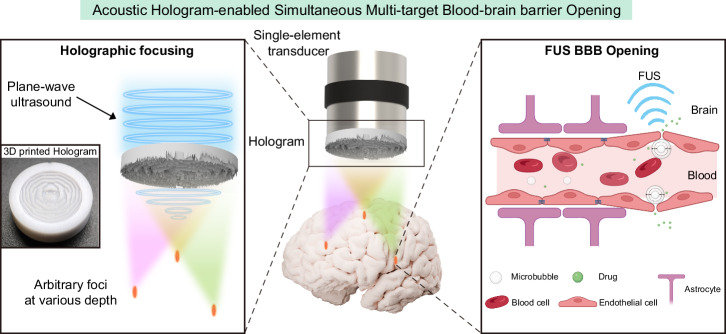
The concept of acoustic hologram-enabled simultaneous multi-target BBB opening (AH-SiMBO). A single-element plane-wave transducer coupled with an acoustic hologram can achieve simultaneous multi-target BBB opening. BBB blood-brain barrier.

In this study, we developed multiple acoustic holograms using the IASA and cost-effective 3D printing to create single and multiple foci at various depths and brain regions. We verified that the designed acoustic hologram coupled with a single-element plane-wave transducer can effectively generate focused acoustic fields for BBB opening through numerical simulations and hydrophone-based measurements. Additionally, lateral and axial beam steering capability is demonstrated by simulations and measurements, and further validated in vivo. We designed a biplane multi-target hologram that constrained focal pressure across different foci with less than 10% variation, and successfully achieved consistent multi-target BBB opening in the bilateral hippocampus (targeting two regions) and medial septum (MS) of mice in a single sonication session. We validated the BBB opening through T1-weighted MRI and fluorescence imaging. Post-treatment assessments, including T2-weighted MRI and histological analyses, showed no adverse effects. This innovative approach is time-efficient and cost-effective with broad applications in brain drug delivery (Fig. 1).

## Results

### Characterization of single-target holograms

**Fig. 2:**
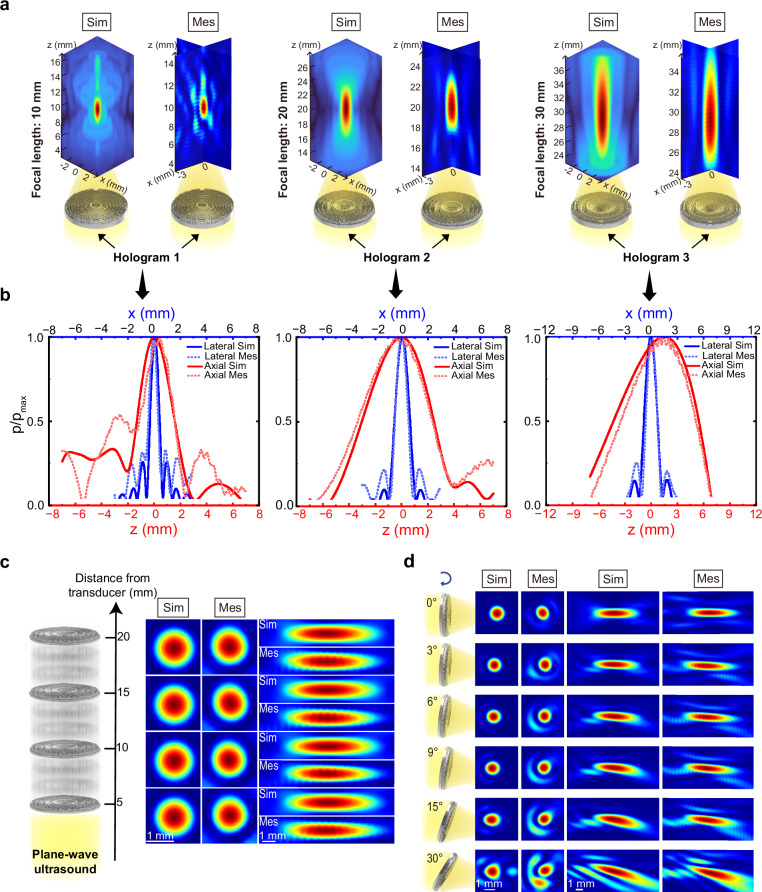
Characterization of holograms by simulation and measurement. **a** The simulated and measured acoustic field of holograms 1, 2 and 3 with different focal lengths of 10, 20, and 30 mm, respectively. **b** Profiles of holograms 1, 2, and 3 (from left to right) along the lateral and axial direction. **c** The simulated and measured acoustic field of the hologram 2 translated along the beam direction by 5, 10, 15, and 20 mm. **d** The simulated and measured acoustic field of the hologram 2 rotated by 0°, 3°, 6°, 9°, 15° and 30°. Sim simulation, Mes measurement.

To characterize the focusing properties of acoustic holograms based on our design method, we designed and fabricated holograms with focal lengths of 10, 20, and 30 mm, labeled as hologram 1, 2 and 3. The holograms with different focal lengths demonstrated excellent focusing performance in both simulation and experimental measurement, with actual focal lengths closely matching the design specifications (Fig. 2a and Supplementary Fig. 1). Holograms with a larger focal length resulted in a larger focal size (lateral full width at half maximum (FWHM): 0.9 mm for hologram 1, 1.3 mm for hologram 2 and 1.7 mm for hologram 3; axial FWHM: 3.4 mm for hologram 1, 6.0 mm for hologram 2 and 13.1 mm for hologram 3) (Fig. 2b and Table 1). The focusing performance of fabricated holograms were characterized in the acoustic measurement tank. Hologram 2 demonstrated the closest focusing performance to the simulation with lateral and axial FWHM of 1.08 mm and 5.88 mm, respectively (Fig. 2b and Table 1). Compared to simulation, hologram 1 and hologram 3 produced a smaller focal region (lateral FWHM: 0.78 mm for hologram 1, 1.37 mm for hologram 3; axial FWHM: 2.72 mm for hologram 1, 11.68 mm for hologram 3) (Fig. 2b and Table 1).

**Table 1:** The simulated and measured FWHM along the lateral and axial direction of holograms with different focal length

Hologram	Hologram 1			Hologram 2			Hologram 3		
Focal Length	10 mm			20 mm			30 mm		
	Sim (mm)	Mes (mm)	Diff	Sim (mm)	Mes (mm)	Diff	Sim (mm)	Mes (mm)	Diff
Lateral FWHM	0.90	0.78	13.33%	1.30	1.08	16.92%	1.70	1.37	19.41%
Axial FWHM	3.40	2.72	20.00%	6.00	5.88	2.00%	13.10	11.68	10.84%
Actual focal length	10.7	10.8	0.93%	20.9	21.0	0.48%	30	30.20	0.67%

*Diff* difference.

To test the beam steering capability of the holograms, we translated hologram 2 by 5, 10, 15, and 20 mm along the beam direction, and rotated hologram 2 by 3°, 6°, 9°, 15° and 30°. In both simulation and experimental measurement, the focal size and focal length remained almost unchanged after translation (Fig. 2c and Table 2). The distance between the focal spot and transducer surface increased by the same 5, 10, 15, and 20 mm, successfully achieving axial beam steering. Lateral beam steering was also achieved (Fig. 2d). Compared to the results with no rotation, rotating the hologram by 3°, 6° and 9° had little impact on the focusing performance, whereas rotating by 15° and 30° manipulated the focal shape and beam direction (Fig. 2d). When the rotation angle was less than 9°, the effect on the lateral and axial FWHM was minimal. However, at rotation angles of 15° and 30°, the measured lateral FWHM increased and the axial FWHM decreased due to the change of beam direction (Fig. 2d and Table 3).

**Table 2:** The simulated and measured FWHM along the lateral and axial direction of holograms with different translation distances

Distance	5 mm		10 mm		15 mm		20 mm		
	Sim (mm)	Mes (mm)	Sim (mm)	Mes (mm)	Sim (mm)	Mes (mm)	Sim (mm)	Mes (mm)	Average diff
Lateral FWHM	1.30	1.13	1.30	1.15	1.30	1.16	1.30	1.17	11.35%
Axial FWHM	6.10	6.09	6.00	6.07	6.10	5.94	6.30	5.98	2.26%
Actual focal length	20.8	20.9	20.8	20.9	20.8	20.8	20.9	20.8	0.36%

*Diff* difference.

**Table 3:** The simulated and measured FWHM along the lateral and axial direction of holograms with different rotation angles

Angle	3°		6°		9°		15°		30°		
	Sim (mm)	Mes (mm)	Sim (mm)	Mes (mm)	Sim (mm)	Mes (mm)	Sim (mm)	Mes (mm)	Sim (mm)	Mes (mm)	Average diff
Lateral FWHM	1.30	1.09	1.30	1.09	1.30	1.11	1.50	1.15	1.50	1.24	17.52%
Axial FWHM	5.40	6.03	5.40	6.07	5.20	5.58	4.90	5.34	4.20	4.90	11.41%

*Diff* difference.

### Characterization of biplane multi-target hologram

**Fig. 3:**
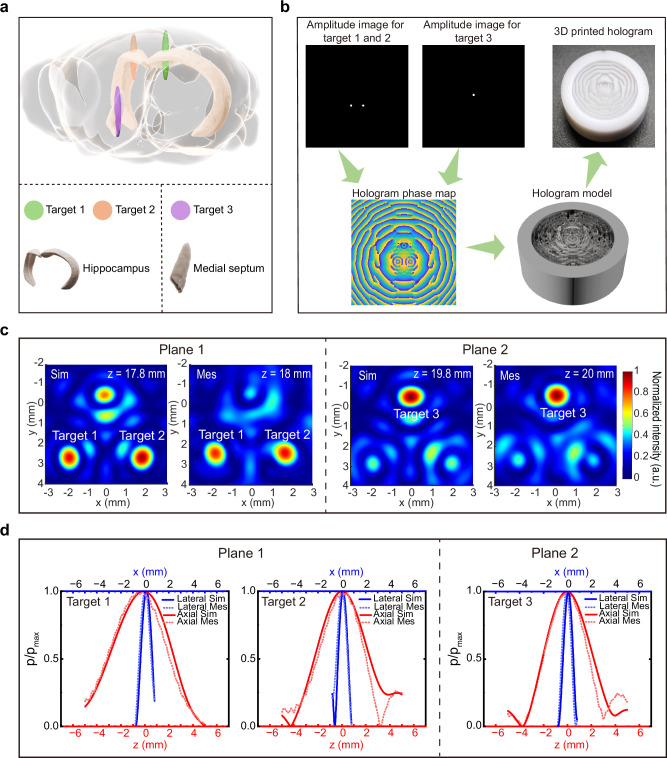
Design and characterization of biplane multi-target hologram. **a** Schematic diagram of the three targets in the mouse brain: bilateral hippocampus and MS. **b** The workflow for the design and production of the biplane multi-target hologram. **c** The simulated and measured acoustic field distribution of targets 1, 2 and 3 at two planes. **d** Profiles of different targets along the lateral and axial direction. MS medial septum, Sim simulation, Mes measurement.

We designed and fabricated a biplane multi-target hologram to simultaneously sonicate the bilateral hippocampus (targeting two regions), and MS brain regions, which were located on different planes (Fig. 3a). Phase map of the hologram was calculated based on the target amplitude image by IASA (Fig. 3b). The biplane multi-target hologram was then reconstructed into a model by calculating the thickness distribution from the phase map and then printed out with a 3D printer (Fig. 3b). We simulated and measured the acoustic field generated by the biplane multi-target hologram. Without amplitude modulation, the focal pressure variation across different foci on different planes in simulation was around 20% (Supplementary Fig. 2). To solve the issue of amplitude variation across different foci, we performed amplitude modulation by assigning different weights to each focus during the designing process and successfully constrained the focal pressure difference within 10% (Fig. 3c). In simulation, two symmetrically distributed focal points (target 1 and target 2) were observed with normalized amplitudes of 0.926 and 0.997, located at (*x*, *y*) = (−1.8, 2.6) mm and (*x*, *y*) = (1.7, 2.6) mm, respectively, at a distance of 17.8 mm from the single-element plane-wave transducer (Fig. 3c). In the second plane at a distance of 19.8 mm from the transducer, target 3 with a normalized amplitude of 1 was observed at (*x*, *y*) = (−0.3, −0.5) mm (Fig. 3c). The measurement results were consistent with the simulation with normalized amplitude of 0.87, 0.89, 1 and location of (*x*, *y*) = (−1.7, 2.4), (1.7, 2.4) and (0.1, −0.5) for target 1, 2, and 3, respectively (Fig. 3c). The high consistency between the measured focal size and the designed focal size (lateral FWHM: 1.1 mm vs. 0.97 mm for target 1, 0.87 mm vs. 1.07 mm for target 2, and 0.96 mm vs. 1.08 mm for target 3; axial FWHM: 6.57 mm vs. 5.69 mm for target 1, 4.68 mm vs. 3.69 mm for target 2, and 4.54 mm vs. 3.45 mm for target 3), demonstrated its great biplane multi-target focusing capability (Fig. 3d).

### Feasibility of AH-SiMBO in mice

**Fig. 4:**
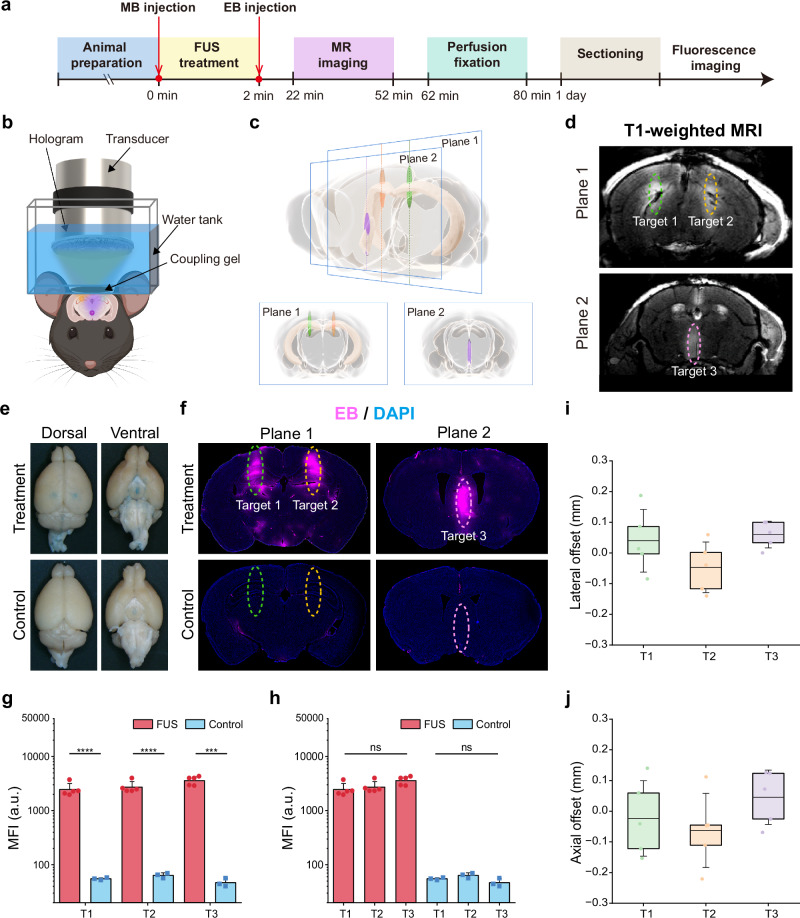
Hologram-enabled simultaneous biplane multi-target BBB opening in mice. **a** Experimental timeline of AH-SiMBO. **b** Schematic diagram showing the single-element plane-wave transducer coupled with a biplane multi-target hologram for FUS BBB opening. **c** 3D and 2D images showing the three target regions: bilateral hippocampus and MS, visualized on two separate brain slices. **d** Axial T1-weighted MR images showing the three BBB opening regions, marked in colored ellipses. **e** Representative brain photographs showing the EB delivery in treated and control mice. **f** Representative 30 μm thick brain slices displaying leakage of the EB. **g**, **h** MFI given in arbitrary units (a.u.) for EB leakage was quantified using ImageJ for each target region (*n* = 5 mice in the FUS group; *n* = 3 mice in the control group). Lateral (**i**) and axial (**j**) offset between the three actual target positions (T1, T2, T3) and the corresponding designed target positions. Center line, mean; box limits, upper and lower quartiles; whiskers, 1x standard deviation; points, data points. The PNPs were 0.28, 0.30, and 0.33 MPa for targets 1, 2, and 3, respectively. BBB blood-brain barrier, FUS focused ultrasound, MS medial septum, EB Evans Blue, MFI mean fluorescence intensity. Two-way ANOVA with post-hoc Bonferroni test in (**g**, **h**). Data are presented as mean ± SD, ns not significant, ***P < 0.001, ****P < 0.0001.

To evaluate the capability of designed biplane multi-target hologram in targeting selected brain regions, we used this hologram for BBB opening in mice (Fig. 4a). A single-element plane-wave transducer, coupled with the designed biplane multi-target hologram, was positioned over the mouse head in a water tank, with ultrasound gel applied between the mouse head and water tank for coupling (Fig. 4b). Plane-wave ultrasound was transmitted through the multi-target hologram and focused on three specified brain regions (two in hippocampus and one in MS) in the mouse brain (Fig. 4c). Considering the insertion loss of mouse skull^37^, the peak negative pressure (PNP) applied on the three targets were 0.28, 0.30 and 0.33 MPa, respectively. To assess BBB opening, we performed contrast-enhanced T1-weighted MRI of the mouse brain following hologram-enabled BBB opening. T1-weighted MR images showed gadolinium extravasation at three targets in the designed planes (Fig. 4d). The representative dorsal and ventral views of the treated mouse brains show successful Evans Blue (EB) leakage at three targets. And there was no significant EB leakage in the control group (Fig. 4e). Fluorescence image of the brain slices across target 1, 2, and 3 depicted a detailed EB distribution in both bilateral hippocampal brain regions on plane 1 and MS brain region on plane 2, which is in great alignment with the designed BBB opening targets and MRI results (Fig. 4f). In the treatment group, the mean fluorescence intensity (MFI) of EB leakage at target 1, target 2, and target 3 are 2.45 ± 0.576 × 10^8^, 2.72 ± 0.576 × 10^8^ and 3.59 ± 0.527 × 10^8^, respectively. In the control group, the MFI at these focal points is 5.49 ± 0.228 × 10^6^, 6.40 ± 0.551 × 10^6^ and 4.69 ± 0.676 × 10^6^, respectively. The significant differences between the treatment and control groups at each target indicate the successful BBB opening (Fig. 4g, *P* < 0.0001 for T1 and T2, *P* < 0.001 for T3). No significant fluorescence intensity was observed among different target regions within treatment or control group (Fig. 4h). The distance between the actual BBB opening locations and the designed target locations for the three targets are 0.04 ± 0.09 mm, −0.05 ± 0.07 mm, and 0.06 ± 0.04 mm along the lateral direction (Fig. 4i) and −0.02 ± 0.11 mm, −0.06 ± 0.10 mm, and 0.05 ± 0.08 mm along the axial direction (Fig. 4j).

### Safety assessment of AH-SiMBO

**Fig. 5:**
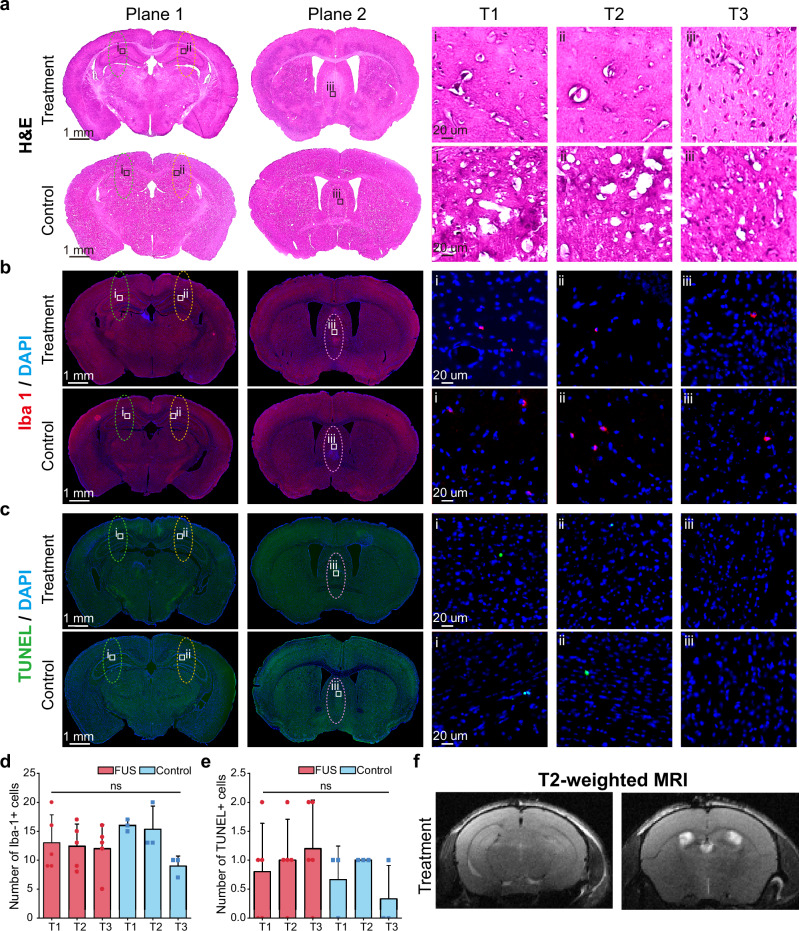
Safety assessment of AH-SiMBO. **a** Representative H&E staining of sections for the treated and the control mice. **b** Representative Iba1 staining sections for the treated and the control mice. **c** Representative TUNEL staining sections for the treated and the control mice. Number of Iba-1 (**d**) and TUNEL (**e**) positive cells in each target region (T1, T2, T3) per slice (*n* = 5 mice in the FUS group; *n* = 3 mice in the control group). Two-way ANOVA with post-hoc Bonferroni test in (**d**, **e**). **f** T2-weighted MR images of the mouse brain following AH-SiMBO. The PNPs were 0.28, 0.30, and 0.33 MPa for targets 1, 2, and 3, respectively. Green, orange and purple ellipses represent targets 1, 2, and 3, respectively. Data are presented as mean ± SD, ns: not significant. BBB blood-brain barrier, FUS focused ultrasound.

To assess the safety of AH-SiMBO, we performed histological analyses to the mouse brain slices, including Hematoxylin and Eosin (H&E) staining (Fig. 5a), Ionized calcium-binding adaptor molecule 1 (Iba1) staining (Fig. 5b) and Terminal deoxynucleotidyl transferase dUTP nick end labeling (TUNEL) staining (Fig. 5c). The representative H&E images showed no cell damage or alternations in brain morphology after AH-SiMBO (Fig. 5a). Immunohistological analysis of mouse brains following AH-SiMBO revealed no significant differences in the number of Iba-1 or TUNEL positive cells compared with control groups (Fig. 5d, e). The T2-weighted images showed no dark areas in the targeted hippocampus and MS region, indicating no edema or hemorrhage occurred during AH-SiMBO (Fig. 5f).

## Discussion

Simultaneous multi-target FUS BBB opening is highly desirable for personalized and targeted therapy^38,39^. In this study, we designed acoustic holograms with both single target and multiple targets, and successfully achieved simultaneous biplane multi-target BBB opening in mice using the designed hologram with a single-element plane-wave transducer. Our study presents several findings: (1) Holograms designed with IASA can produce multiple foci at arbitrary planes while maintaining similar focal pressure; (2) Hologram demonstrated the capability of beam steering; (3) A single-element plane-wave transducer, combined with a hologram, can safely achieve simultaneous multi-target BBB opening at arbitrary planes.

This study presents an approach to designing acoustic holograms using IASA for simultaneous multi-target (≥3) BBB opening at arbitrary planes in animals. The ability to achieve simultaneous multi-target BBB opening holds great therapeutic potential, particularly for treating complex neurological conditions requiring localized drug delivery to multiple brain regions^40^. Current phased-array methods for multi-target BBB opening cannot achieve simultaneity, resulting in temporal delays in drug delivery between targets, which can reduce therapeutic efficacy^41^. Among various beam focusing techniques, hologram stands out due to its low cost, reduced energy loss and without the need for real-time phase correction compared to phased-array systems. Previous studies designed acoustic holograms by time-reversal approach and successfully achieved single-plane dual-target BBB opening in the mouse model^30,32^. However, these studies did not achieve multi-target BBB opening at arbitrary planes, restricting its broader applications. Our study specifically selected three targets on two planes and achieved precise simultaneous BBB opening at the corresponding sites in vivo.

The PNP is a critical acoustic parameter in achieving BBB opening. Previous studies have shown that when the PNP is between 0.3 MPa and 0.45 MPa, FUS combined with microbubbles can successfully induce the BBB opening without causing any cell damage, such as red blood cell extravasations^42,43^. The FUS parameters in the animal experiment of our work were selected based on a prior study^44^. Additionally, previous research has shown that ultrasound at 1.5 MHz undergoes approximately 20% attenuation when passing through the mouse skull^37^. Accordingly, the derated PNP values of our treatment procedure were 0.28, 0.30, and 0.33 MPa. In this study, the spatial peak pulse average intensity (Isppa) and the spatial peak temporal average intensity (Ispta) were 5.6 W cm^−2^ and 56 mW cm^−2^, which are well below the FDA safety guideline (Isppa <190 W cm^−2^, Ispta <720 mW cm^−2^)^45^. Histological analyses confirmed the safety with these parameters (Fig. 5a–e). The uniform PNP among all targets is essential for the consistency and safety of simultaneous multi-target BBB opening. In this study, we successfully controlled the variation of acoustic pressure across different targets within 10% by amplitude modulation between the two planes. No significant difference in MFI was observed among three targets (Fig. 4h). The 10% discrepancy between two different planes may stem from the design process using the IASA, where the hologram’s thickness was not fully considered, potentially influencing the iterative process and causing variations across different foci. Additionally, the variation in attenuation along each focal path, due to the asymmetric distribution of thickness, may also contribute to this discrepancy.

Previous studies found that a direct cholinergic circuit from the MS to the hippocampus was shown to reduce seizures by driving hippocampal somatostatin inhibition^46^. And the activation of cholinergic neurons in the MS enhances acetylcholine release in the hippocampus^47^. In line with these findings, we successfully achieved simultaneous multi-target BBB opening in the MS and bilateral hippocampus brain regions—key areas involved in the treatment of epilepsy, which demonstrated the potential of hologram-enabled FUS to deliver simultaneous targeted therapies for neurological conditions requiring multi-target intervention.

The results of translation and rotation tests demonstrated the designed acoustic hologram’s capability of beam steering in both axial and lateral direction (Fig. 2c, d). We further validated the beam steering capabilities of our hologram through in vivo experiments (Supplementary Fig. 3). Axial translation of the hologram by 6 mm induced a precisely matched 6 mm shift in the BBB opening site, as evidenced by displacement of the EB leakage region (Supplementary Fig. 3a). Rotational control was similarly confirmed via a 30° lateral hologram rotation, which produced a corresponding angular shift in the BBB opening site within the coronal plane (Supplementary Fig. 3b). The key advantage of beam steering lies in its ability to precisely control both the focal points and beam direction. In comparison, phased-array systems can achieve beam steering using techniques such as liquid lens^48^ or electronic beamforming^49^. However, both two methods have the disadvantage of the complex lens design process and limited beam steering range. In contrast, holograms offer greater flexibility, enabling more efficient and versatile beam steering across a wider range of angles and depths. The beam steering capability of acoustic holograms allows dynamic beam manipulation during treatment, enabling real-time adjustments and increasing clinical adaptability, especially for multi-target BBB opening and large-volume or irregularly shaped thermal ablation^50^.

The IASA was used to design holograms in this study. Previous studies successfully used a time-reversal approach to design holograms, achieving symmetric bifocal BBB opening in both mouse and non-human primate models^30,31^. However, time-reversal-based holographic focusing has several limitations: (1) Limited targeting flexibility—Time-reversal relies on recorded wavefront and lacks precise multi-focal control, making it challenging for three-dimensional multi-focal targeting. (2) Lack of amplitude control—Time-reversal does not explicitly modulate focal amplitudes, leading to energy imbalances that hinder precise and safe BBB opening or neuromodulation across multiple targets. (3) Weak sidelobe suppression—Time-reversal relies on precise experimental wavefront recordings rather than iterative optimization, leading to suboptimal sidelobe suppression and limited adaptability. A deep learning-based approach (i.e., autoencoder-like architecture called HU-Net) has also been used for fast and accurate acoustic hologram generation^51^. While promising, this approach usually requires a large amount of high-quality labeled data for training to ensure the generalization of the model. Compared to these approaches, IASA stands out for its ability to achieve arbitrary multi-focal targeting in three-dimensional space, making it particularly valuable for treating anatomically distributed pathologies. Additionally, its amplitude modulation capability ensures <10% pressure variation across foci, enabling uniform bioeffects such as consistent BBB opening in multi-target interventions. Furthermore, its scalability enables easy adaptation to larger apertures, and its capacity for encoding detailed phase information offers greater flexibility in designing complex and intricate holograms.

There are several limitations in this study. First, consideration of the skull-induced phase aberration is warranted for hologram design when adapting to humans or large animals. In future studies, CT scans of the skull will be incorporated into the iterative process of IASA to compensate for phase aberrations induced by the skull heterogeneity. Second, our current holographic system lacks acoustic cavitation detection capability. Real-time feedback control of the FUS energy based on harmonic emissions is warranted to ensure consistent and safe BBB opening across different targets and subjects^12,52^. Third, during the cleaning process of the printed holograms, the supporting material has to be dissolved in oil at 55 °C. This step may cause deformation of the hologram’s microstructures, potentially affecting focusing performance and position offset.

Overall, we designed holograms by IASA and fabricated them via 3D printing technology to generate single or multiple foci at different depths and regions. Lateral and axial beam steering capability is demonstrated by simulations, measurements and in vivo validation. We successfully achieved simultaneous biplane multi-target (≥3) BBB opening in the bilateral hippocampus (targeting two regions) and MS (targeting one region) of mice in a single sonication session. Contrast-enhanced T1-weighted MRI and fluorescence intensity statistics confirmed the successful BBB opening. The post-treatment T2-weighted MRI and histological analyses revealed no signs of edema, hemorrhage, cellular damage, or inflammation, indicating that the procedure is safe and well-tolerated. Our results demonstrated the feasibility, efficiency, and safety of AH-SiMBO, underscoring its potential as a time-efficient and cost-effective approach for multi-target brain disorder treatment.

## Methods

### Hologram design algorithms

Acoustic holograms were designed by the IASA. The desired wavefront can be reconstructed in a specific plane by calculating and optimizing the hologram’s phase distribution in an iterative process. Due to the restriction of the diffraction limit, we selected 100 μm as the pixel size, which is about one-tenth of the wavelength. The phase distribution was obtained after 100 iterations. The procedures of the IASA are as follows.

The acoustic pressure wave from the transducer was defined as a summation of plane waves.

1 $${p}_{0}(x,y,z)=|{p}_{0}|(x,y,z=0){e}^{j\Delta \phi (x,y,z)}.$$

where$\,\left|{p}_{0}\right|$ and $\Delta {{\rm{\phi }}}$ indicate the amplitude and phase of the ultrasound wave. The acoustic angular spectrum at the initial position of the acoustic hologram was obtained by a two-dimensional Fourier transform.

2 $$p({k}_{x},{k}_{y},z)=\int {\int }_{-\infty }^{+\infty }p(x,y,z){e}^{-j({k}_{x}x+{k}_{y}y)}dxdy.$$

Multiplying the angular spectrum at *z* = 0 with the propagation function yielded the angular spectrum of the acoustic wave in the target plane (*z* = *z*).

3 $$p({k}_{x},{k}_{y},z)={p}_{0}({k}_{x},{k}_{y},0)H({k}_{x},{k}_{y},z).$$

The angular spectrum of the acoustic wave in the target plane was inverted by a two-dimensional inverse Fourier transform to obtain the pressure distribution at z in the target plane.

4 $$p({{\rm{x}}},{{\rm{y}}},{{\rm{z}}})=\frac{1}{4{\pi }^{2}}\int {\int }_{-\infty }^{+\infty }p({k}_{x},{k}_{y},z){e}^{j({k}_{x}x+{k}_{y}y)}d{k}_{x}d{k}_{y}.$$

The quality of the modulation of the reconstructed acoustic field was evaluated while keeping the phase as a constraint. The angular spectrum in the target plane was back-propagated to the acoustic hologram plane after resetting the amplitude.

5 $${p}_{0}\left({k}_{x},{k}_{y},0\right)=p({k}_{x},{k}_{y},{z})H({k}_{x},{k}_{y},-{z}).$$

The amplitude at each focus for each plane was calculated individually, and the amplitude weights were adjusted according to the amplitude distribution at these points. Iterations were performed until the amplitude difference across all foci was reduced to less than 5%. The phase distribution of the acoustic field was obtained by summing the complex pressure distribution. Subsequently, the transmission coefficient *T* and the phase distribution were calculated based on the material properties. The thickness of each pixel of the corresponding acoustic metasurface was determined after accounting for phase transitions, and the amplitudes were recalibrated and re-integrated into the iterative process.

6 $${{\rm{T}}}=\frac{4{Z}_{1}{Z}_{2}}{\left({Z}_{1}-{Z}_{2}\right)\left({Z}_{2}-{Z}_{3}\right){e}\,^{j\phi }+\left({Z}_{1}+{Z}_{2}\right)\left({Z}_{2}+{Z}_{3}\right)},$$

where ${Z}_{1}$,$\,{Z}_{2}$,$\,{Z}_{3}$ represent the acoustic impedance values of different materials.

When the iteration reached convergence, the iteration was stopped, and the phase distribution of the final hologram was obtained. The thickness distribution of the hologram was converted from the phase distribution.

### Acoustic field simulation using K-wave

For the acoustic field simulation, we applied a pseudo-spectral full-wave method incorporating k-space dispersion correction^53^. An isotropic numerical grid was used with a spatial step of $\Delta {{\rm{h}}}=100\,{{\rm{\mu }}}{{\rm{m}}}$, providing a spatial sampling of 5 grid points per wavelength for a frequency of 1.5 MHz. The grid contained over 300 million points to ensure high accuracy. The temporal step $\Delta {{\rm{t}}}$ was selected according to the wave speed in the medium, ensuring a stable Courant-Friedrichs-Lewy (CFL) number^54^. The primary simulation medium was water, with a sound speed of 1480 m s^−1^ and a density of 1000 kg m^−3^. Additionally, a hologram with a sound speed of 2290 m s^−1^and a density of 1030 kg m^−3^ was modeled. The properties of the hologram were applied using a thickness distribution matrix resized to match the grid dimensions. Acoustic pressure and maximum pressure values were recorded across the simulation domain by the sensor.

### Hologram fabrication

The thickness map obtained after iteration was reconstructed by COMSOL (COMSOL Multiphysics 6.2, COMSOL, Stockholm, Sweden). The designed hologram was fabricated by a 3D printer (ProJet MJP2500, 3D SYSTEMS, Rock Hill, SC, USA). The printing material, Visjet M2R-WT, had a sound speed of 2290 m s^−1^, a density of 1030 kg m^−3^ and an absorption coefficient of 4.6 dB cm^−1^ at 1.5 MHz. After printing, the object was immersed in oil for over 12 h to completely dissolve the support material. The cleaned hologram was then obtained. By positioning the hologram in front of a single-element plane-wave transducer, the acoustic distribution in the target plane could be obtained.

### Acoustic field measurement

The 3D-printed acoustic hologram was coupled with a single-element plane-wave transducer (TP1.5P30NF, Guangzhou Doppler Electronic Technologies, Guangzhou, Guangzhou, China). The acoustic field produced by holograms was measured by an ultrasound measurement system (Supplementary Fig. 4). The single-element plane-wave transducer had a center frequency of 1.5 MHz. The transducer was connected with an electrical driving system composed of a signal generator (33500B, Keysight Technologies, Santa Rosa, CA, USA) and a power amplifier (240 L, Electronics & Innovation, Rochester, NY, USA). The transducer was mounted on a holder connected to the measurement tank filled with degassed and deionized water. The acoustic field was measured by a hydrophone (NH0500, Precision Acoustics, Dorchester, Dorset, UK) mounted on a 3D-translation stage. After amplification by a preamplifier and coupling via a direct current coupler (DCPS0188, Precision Acoustics, Dorchester, Dorset, UK), the electrical signal is stored by the oscilloscope (DSOX3012A, Keysight Technologies, Santa Rosa, CA, USA). The lateral and axial pressure fields centered on the focus were measured with a step size of 0.2 mm (one-fifth of the wavelength). The pressure fields were normalized to the maximum pressure. To test the focusing stability of the hologram, we measured the acoustic field after translating the hologram by 5, 10, 15, and 20 mm. Additionally, to study the impact of rotation on the focusing performance of holograms, we measured the acoustic field after rotating the hologram by 3°, 6°, 9°, 15° and 30°. The translation and rotation were implemented by a translation stage (LSSZ-03-05, Jiangxi Liansheng Technology, Nanchang, Jiangxi, China) and a rotary mount (LSJC3-12-12, Jiangxi Liansheng Technology, Nanchang, Jiangxi, China). The translation stage provided a movement range of 25 mm in each of the three directions, while the rotary mount allowed for 360-degree rotation.

### Hologram-enabled BBB opening procedure

All animal experimental procedures were conducted following the guidelines of the National Institutes of Health and approved by the Institutional Animal Care and Use Committee of ShanghaiTech University. Adult male C57BL/6 mice between 6–10 weeks old of age (*n* = 8, Jihui, Shanghai, China), weighing from 20 to 24 g, were used in this study. All mice were randomly divided into treatment group (*n* = 5) and control group (*n* = 3). Experiments were performed in a randomized order. All mice were housed at a temperature of 25 °C with a 12 h light-dark cycle. Anesthesia was induced by isoflurane (5%) and oxygen (0.8 L min^−1^). The absence of a pedal reflex confirmed successful induction, and isoflurane was then decreased and adjusted between 1.5–2% to maintain anesthesia without producing gasping from low oxygenation. The mice’s head was fixed by a stereotactic frame (Robot Stereotaxic, NEUROSTAR, Tübingen, Baden-Württemberg, Germany) using ear and bite bars to immobilize the head. A heating pad with a temperature probe was used to maintain the body temperature between 36–37 °C. In the treatment group, hair over the mice’s head was removed with depilation cream. A precise skin incision on the mouse head was made to expose the cranial bregma and lambda landmarks, while the skull remained fully intact. Microbubbles (SonoVue, Bracco, Milan, Lombardy, Italy) were administrated through a tail vein catheter. The microbubble solution was activated according to the manufacturer’s protocol. 5 mice received 2 min of FUS exposure after receiving an i.v. bolus of microbubble solution (2.5 μL g^−1^). FUS exposure (PNPs of target 1, target 2 and target 3: 0.35, 0.37, and 0.41 MPa; pulse length: 10 ms; pulse repetition frequency: 1 Hz; number of pulses: 120) was delivered right after microbubble administration. After FUS exposure, EB dye (2%, 3.33 mL kg^−1^) was injected immediately through the tail vein catheter to confirm BBB opening. The control group only received EB injected through the tail vein.

### MRI procedure

The mice were scanned using a small-animal 9.4 T MRI system (Bruker, Billerica, MA, USA) at 60 min after FUS exposure, anesthetized with 0.75–1.5% isoflurane and placed in a prone position with the head located at the center of the coil. The MR parameters were as follows: axial T2WI turbo-spin echo (TSE) (repetition time [TR]/echo time [TE]: 2005.071/35 ms; flip angle: 90°; number of excitations [NEX]: 8; in-plane resolution: 0.1 × 0.1 mm; slice thickness: 0.3 mm; inter-slice gap: 0.3 mm; receiver bandwidth: 81967.2 Hz); axial T1WI fast gradient echo (Fast GRE) (repetition time [TR]/echo time [TE]: 325.196/3.471 ms; flip angle: 70°; number of excitations [NEX]: 5; in-plane resolution: 0.067 × 0.067 mm; slice thickness: 0.3 mm; inter-slice gap: 0.3 mm; receiver bandwidth: 58823.5 Hz). T1-weighted MRI scans were acquired before and after intravenous administration of 0.1 mol L^−1^ gadoteric acid contrast agent (BidePharm, Shanghai, China). T2-weighted scans were acquired before intravenous administration for assessing safety outcomes of edema and hemorrhage.

### Mouse brain fixation and sectioning

Transcardiac perfusion was performed with saline, followed by 10% paraformaldehyde. The tissues were post-fixed in 10% paraformaldehyde for 24 h and then immersed in 30% sucrose until they sank to the bottom. The tissues were subsequently sectioned using a freezing microtome (CryoStar NX50 OP, Thermo Fisher Scientific, Waltham, MA, USA). The fixed brain samples were sectioned at 10 μm for hematoxylin & eosin (H&E) staining and 30 μm for Iba1 staining and TUNEL staining. The sections were mounted on glass slides and stored at 4 °C until further use.

### H&E staining

We performed H&E staining to evaluate the effect of FUS on cellular morphology in mouse brain tissue. Tissue sections were stained with hematoxylin and eosin reagent according to the protocol. The sections were immersed in the Hematoxylin Stain Solution (60502ES60, Yeasen Biotechnology, Shanghai, China) for 30 s, rinsed under running water for approximately 60 s, followed by incubation in Eosin Stain Solution (0.5%) (S0186, Beijing Biosynthesis Biotechnology, Beijing, China) for 10 s, and then rinsed again for about 30 s. Images of the H&E-stained sections were captured using an Olympus microscope (Olympus, Tokyo, Japan) with a 20X objective.

### Iba1 staining

For immunofluorescence staining, tissue sections were washed 5 times in PBS (1×) (MA0015-500 ml, Dalian Meilun Biotechnology, Dalian, Liaoning, China), with each wash lasting 5 min. The sections were then fixed in 4% paraformaldehyde (30525-89-4, Sinopharm Chemical Reagent, Shanghai, China) at room temperature for 15 min, followed by another five PBS (1×) washes. To increase permeability, the sections were permeabilized with Triton X-100 (P0096-100ml, Beyotime Biotechnology, Shanghai, China) for 15 min, and then rinsed again with PBS (1×) five times. Next, the sections were blocked using a rapid blocking solution and incubated at room temperature for 15 min. Primary antibody, Rabbit Anti-Iba1 (019-1974, FUJIFILM Wako Pure Chemical Corporation, Osaka, Japan), was diluted at a ratio of 1:1000 in QuickBlock™ Primary Antibody Dilution Buffer for Immunol Staining (P0262, Beyotime Biotechnology, Shanghai, China), was applied to fully cover the brain tissue sections, which were then incubated overnight at 4 °C in the dark. To maintain moisture during incubation, a small amount of degassed and deionized water was added to the staining chamber. The following day, the sections were washed three times with PBS (1×) for 5 min each. Afterward, the sections were incubated at room temperature for 1 h in the dark with a fluorochrome-conjugated secondary antibody. The secondary antibody, Alexa Fluor 555-labeled Donkey Anti-Rabbit IgG (H + L) (A0453, Beyotime Biotechnology, Shanghai, China), was diluted at a ratio of 1:400 in Beyotime QuickBlock™ Secondary Antibody Dilution Buffer for Immunofluorescence (P0265, Beyotime Biotechnology, Shanghai, China). The sections were then washed five times with PBS (1×), for 5 min each. Finally, the brain tissue sections were mounted using an appropriate mounting medium. Iba1 staining results were observed using an Olympus microscope (Olympus, Tokyo, Japan) with a 10X objective.

### TUNEL staining

TUNEL was performed to evaluate cell death. The sections were washed five times in PBS (1×) (MA0015-500ml, Dalian Meilun Biotechnology, Dalian, Liaoning, China), with each wash lasting 5 min. Subsequently, the sections were fixed in 4% Paraformaldehyde (30525-89-4, Sinopharm Chemical Reagent, Shanghai, China) at room temperature for 15 min, followed by five additional washes with PBS (1×). Next, the sections were permeabilized with Triton X-100 (P0096-100ml, Beyotime Biotechnology, Shanghai, China) for 15 min to enhance permeability, after which they were washed five more times with PBS (1×). The One Step TUNEL Apoptosis Assay Kit (C1086, Beyotime Biotechnology, Shanghai, China) was added, and the sections were incubated at room temperature in the dark for 1 h. After incubation, the sections were rinsed with PBS (1×) three times for 5 min each. An appropriate amount of diluted DAPI (1:400, C1002, Beyotime Biotechnology, Shanghai, China) staining solution was then applied to cover the brain tissue sections. The TUNEL staining results were observed using an Olympus microscope (Olympus, Tokyo, Japan) with a 10X objective.

### Fluorescence intensity analysis

MFI quantification in arbitrary units (a.u.) for EB leakage was performed using ImageJ. Three tissue sections were analyzed per mouse. And for each section, fluorescence intensity was measured at three target sites. The minimum and maximum intensity thresholds in ImageJ were set consistently across all samples.

### Statistics

Statistical analyses were performed using GraphPad Prism 10.3.1. The Gaussian distribution was determined using the Shapiro-Wilk test. A two-way analysis of variance (ANOVA) followed by Bonferroni’s multiple comparison test was employed. Data are presented as mean ± SD in both the figures and the main text. For all comparisons, statistical significance was defined as *P* < 0.05. Schematic illustrations were created using Origin 2023b.

## Supplementary information

Supplementary Information

## Data Availability

The data that support the findings of this study are available from the corresponding author upon reasonable request.

## Code availability

The code related to the findings of this study is available from the corresponding author upon reasonable request.
