# Supplementary material for: Acoustic hologram-enabled simultaneous multi-target blood-brain barrier opening (AH-SiMBO)
Source: Commun Eng. 2025 Jun 2;4:99. doi: 10.1038/s44172-025-00428-z (PMC12130547; doi:10.1038/s44172-025-00428-z)
Supplement: Supplementary file 1 — Supplementary Information [file 44172_2025_428_MOESM1_ESM.pdf]

Acoustic Hologram-enabled Simultaneous Multi-Target Blood-Brain Barrier Opening (AH-SiMBO)

Xinya Yao[1,2], Xiangkun Piao[1,2], Shulong Hong[3], Chenyu Ji[1,2], Mingyu Wang[1,2], Yan Wei[1,2], Zhouyang Xu[1,2], Jia-Ji Pan[1,2], Yanbo Pei[3,4,5*], and Bingbing Cheng[1,2*]

AFFLIATIONS

[1]Translational Research in Ultrasound Theranostics Laboratory, School of Biomedical Engineering, ShanghaiTech University, Shanghai, 201210, China

[2]State Key Laboratory of Advanced Medical Materials and Devices, ShanghaiTech University, Shanghai, 201210, China

[3]Institute of Modern Optics, School of Physics, Harbin Institute of Technology, Harbin, 150001, China

[4]Key Laboratory of Micro-Nano Optoelectronic Information System of Ministry of Industry and Information Technology, Harbin Institute of Technology, Harbin, 150001, China

[5]Key Laboratory of Micro-Optics and Photonic Technology of Heilongjiang Province, Harbin Institute of Technology, Harbin, 150001, China

[*]Authors to whom correspondence should be addressed: chengbb@shanghaitech.edu.cn and peiyanbo@hit.edu.cn.

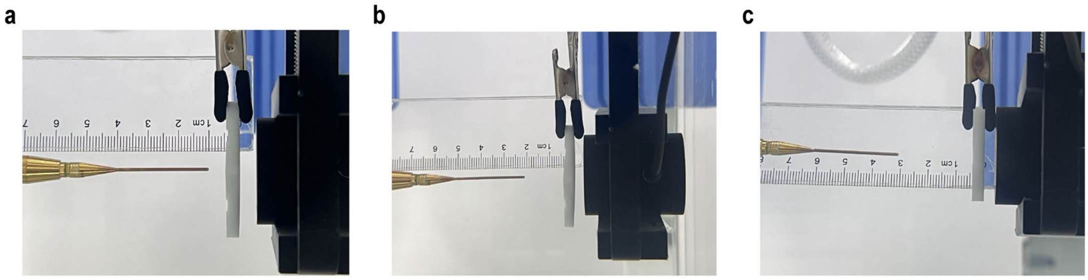

Supplementary Figure. 1 | Focal length of different holograms. Measured focal length of holograms with designed focal length of 10 mm (**a**), 20mm (**b**) and 30 mm (**c**).

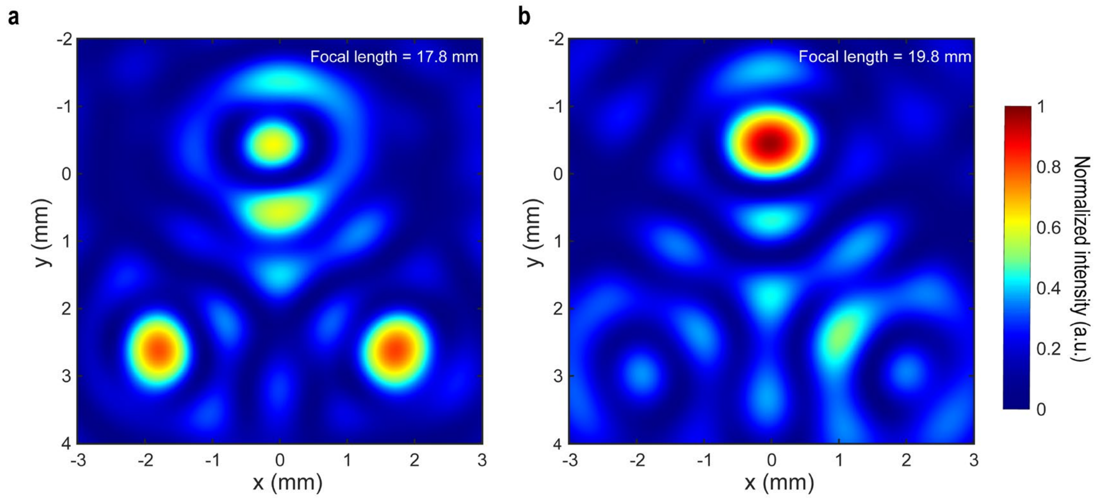

Supplementary Figure. 2 | The simulated acoustic field distribution in different planes without amplitude modulation. a Two foci at the plane with the focal length of 17.8 mm. **b** One focus at the plane with the focal length of 19.8 mm.

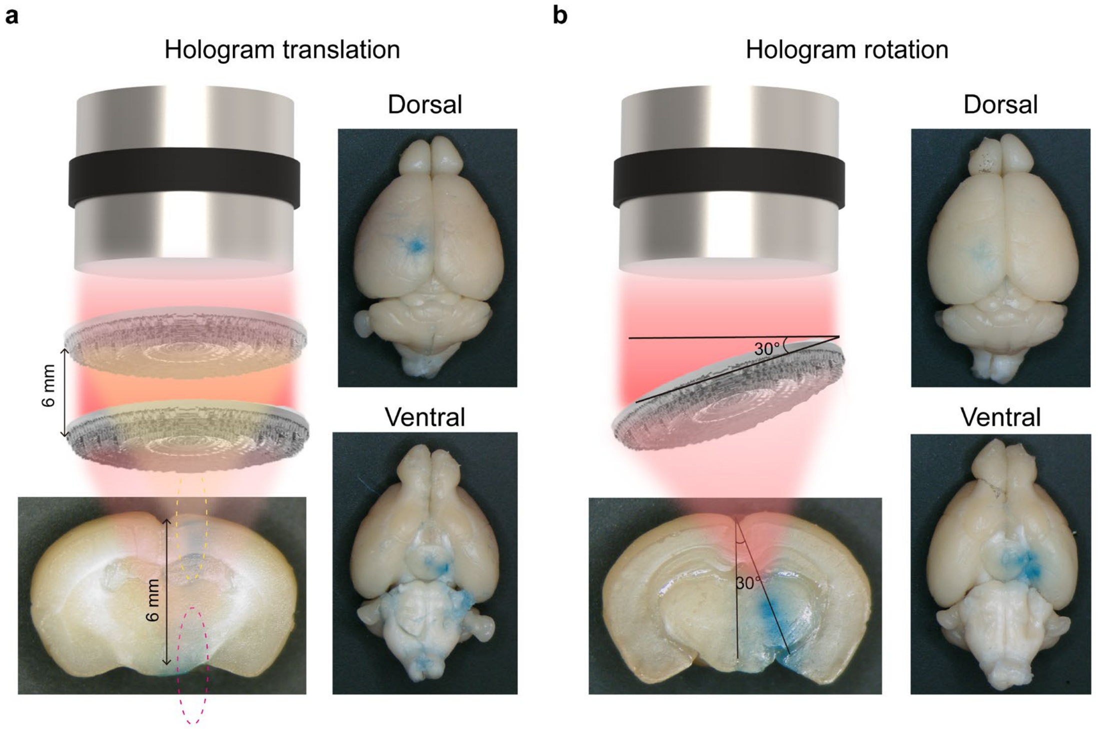

Supplementary Figure. 3 | The beam steering capability validation in *vivo*. a Demonstration of axial beam steering capability through a 6 mm shift of the hologram. **b** Demonstration of lateral beam steering capability through a 30° rotation of the hologram.

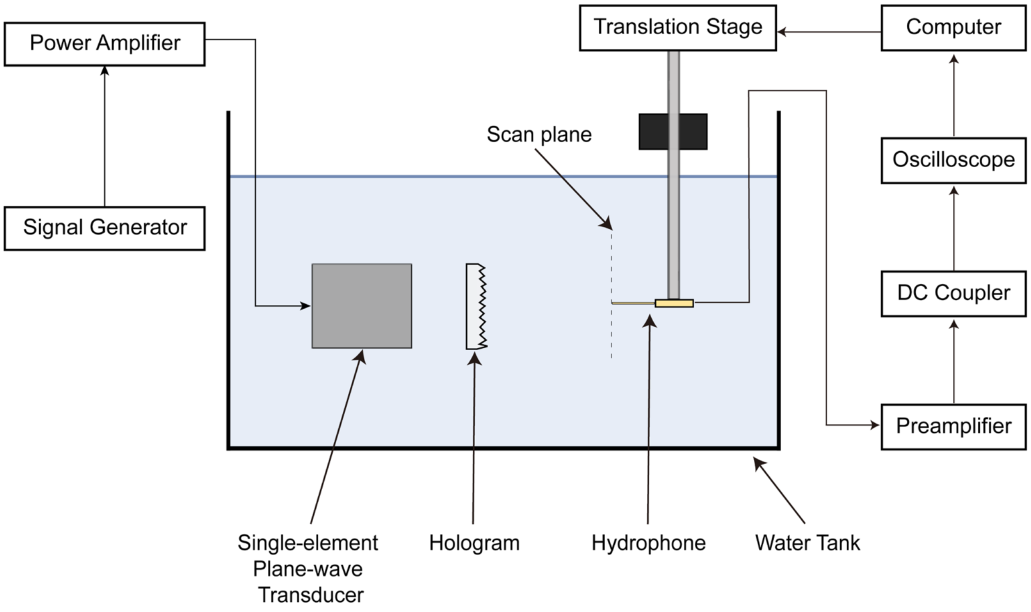

Supplementary Figure. 4 | The acoustic measurement system. Experiment setup for acoustic field characterization.
